# Curation of the AMRFinderPlus databases: applications, functionality and impact

**DOI:** 10.1099/mgen.0.000832

**Published:** 2022-06-08

**Authors:** Michael Feldgarden, Vyacheslav Brover, Boris Fedorov, Daniel H. Haft, Arjun B. Prasad, William Klimke

**Affiliations:** ^1^​ National Center for Biotechnology Information, U.S. National Library of Medicine 8600 Rockville Pike, Bethesda MD, 20894, USA

**Keywords:** curation, antimicrobial resistance, genomics

## Abstract

Antimicrobial resistance (AMR) is a significant public health threat. Low-cost whole-genome sequencing, which is often used in surveillance programmes, provides an opportunity to assess AMR gene content in these genomes using *in silico* approaches. A variety of bioinformatic tools have been developed to identify these genomic elements. Most of those tools rely on reference databases of nucleotide or protein sequences and collections of models and rules for analysis. While the tools are critical for the identification of AMR genes, the databases themselves also provide significant utility for researchers, for applications ranging from sequence analysis to information about AMR phenotypes. Additionally, these databases can be evaluated by domain experts and others to ensure their accuracy. Here we describe how we curate the genes, point mutations and blast rules, and hidden Markov models used in NCBI’s AMRFinderPlus, along with the quality-control steps we take to ensure database quality. We also describe the web interfaces that display the full structure of the database and their newly developed cross-browser relationships. Then, using the Reference Gene Catalog as an example, we detail how the databases, rules and models are made publicly available, as well as how to access the software. In addition, as part of the Pathogen Detection system, we have analysed over 1 million publicly available genomes using AMRFinderPlus and its databases. We discuss how the computed analyses generated by those tools can be accessed through a web interface. Finally, we conclude with NCBI’s plans to make these databases accessible over the long-term.

## Significance as a BioResource to the community

AMR gene-detection tools are critical for using bacterial genomics to understand the spread of antibiotic resistance. Most AMR gene-detection tools rely on reference databases of nucleotide or protein sequences and collections of models and rules for analysis, which are derived from the literature and often designed to meet specific scientific needs, such as food-borne surveillance and other applied uses. These databases can have significant effects on the success of analytical procedures [30] and also can be reutilized for other research purposes. Here, we describe the curation process for the databases, models and rules used by NCBI’s AMRFinderPlus. We also describe our procedure for making these databases publicly available, as well as user interfaces to interrogate these databases. We also describe how users can access computed AMRFinderPlus results for over 1000000 bacterial isolates in NCBI’s Pathogen Detection system. To maintain relevance and accuracy, we describe possible areas of database improvement and how users can assist and guide our database curation.

## Data Summary

**Fig. 1.**
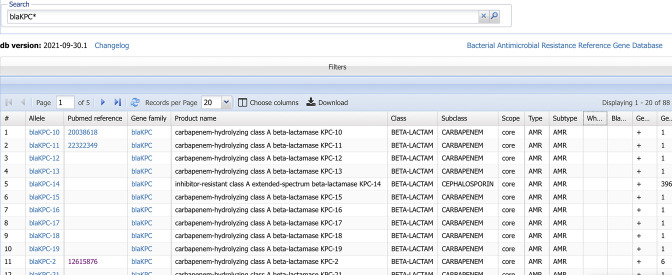
The Reference Gene Catalog. For acquired genes, each row contains the gene symbol, the allele symbol, GenBank and RefSeq nucleotide and protein accessions, phenotype information, and a PubMed citation. For point mutations, each row contains an allele symbol, which is a concatenation of the point mutation and gene symbol of the reference gene, the gene symbol of the reference gene, GenBank and RefSeq nucleotide or protein accessions of the reference sequence, phenotype information and a PubMed citation.

1. Both databases used by AMRFinderPlus can also be downloaded as part of the AMRFinderPlus installation process. AMRFinderPlus is available through GitHub (https://github.com/ncbi/amr), with a wiki that provides additional information on installation and programme use (https://www.github.com/ncbi/amr/wiki). AMRFinderPlus and its databases can be easily installed with Bioconda as described on the AMRFinderPlus wiki.
2. Detailed documentation of database formats and contents is publicly available at https://www.github.com/ncbi/amr/wiki. All database historical releases are retained indefinitely, contain detailed change logs, and are backed up in multiple locations.
3. The databases also are available through the following GUIs:
  - The Pathogen Detection Reference Gene Catalog (https://www.ncbi.nlm.nih.gov/pathogens/refgene/) provides a visualization of acquired genes and point mutations used by AMRFinderPlus, where each row represents an acquired protein sequence or point mutation (see Fig. 1). Core genes are available in BioProject PRJNA313047.
  - The Reference Gene Hierarchy (https://www.ncbi.nlm.nih.gov/pathogens/genehierarchy/) is a web-based view into the hierarchy of genes, families and upstream nodes that NCBI curators use to organize and relate the genes and hidden Markov models (HMMs) in the Reference Gene Catalog and Pathogen Detection Reference HMM Catalog.
  - The Pathogen Detection Reference HMM Catalog (https://www.ncbi.nlm.nih.gov/pathogens/hmm/) is a web-based portal to our curated database of reference HMMs used by AMRFinderPlus in concert with gene sequences in the Pathogen Detection Reference Gene Catalog to identify antimicrobial resistance (AMR) genes as well as some stress resistance and virulence genes.
4. Computed analyses by AMRFinderPlus on the over 1,000,000 isolates in NCBI’s Pathogen Detection system can be found in two different GUIs:
  - The Isolates Browser (https://www.ncbi.nlm.nih.gov/pathogens/isolates/) provides a summary of AMR, stress response and virulence genes for each isolate.
  - The Microbial Browser for Identification of Genetic and Genomic Elements (MicroBIGG-E; https://www.ncbi.nlm.nih.gov/pathogens/microbigge/), displays AMRFinderPlus results for those isolates, which have genomic data deposited in GenBank.

## Introduction

Antimicrobial resistance (AMR) is a significant public health threat, and has been estimated to cause over one million deaths globally [1]. Low-cost whole-genome sequencing, which is often used in surveillance programmes, provides an opportunity to assess AMR gene content in these genomes using *in silico* approaches. These *in silico* approaches can lead to the discovery of novel resistance mechanisms [2], and also can be used to predict resistance phenotypes [3, 4]. A variety of bioinformatic tools have been developed to identify these genomic elements, for purposes ranging from basic research to applied uses such as surveillance and clinical use [4–6]. Most of those tools rely on reference databases of nucleotide or protein sequences and collections of models and rules for analysis.

While the tools are critical for the identification of AMR genes, their underlying databases are a critical component of these tools’ effectiveness; for example, if a gene is missing from a database, it is less likely to be found. The databases themselves also provide significant utility for researchers. For example, researchers interested in drug development can use existing sequence variation to design better drugs, and accessible databases make that process easier. Well-curated databases also contain biological information about those genetic elements they contain, which can be used to understand those elements’ function. Users might use a curated database as the backbone for their own specialized analyses, such as using existing databases for large-scale metagenomic analyses [7]. Also, domain experts and others can evaluate public databases, improving those databases’ accuracy. Thus, it is critical that other researchers are able to access these tools’ databases [8].

Another key element is ongoing curation. Not only should databases be available to others, but ongoing curation, as novel genetic elements related to antimicrobial susceptibility and pathogenesis are routinely discovered, is essential for keeping databases functional and useful. To do this, database curation should involve:

- Gathering novel information.
- Ensuring the reliability of the data.
- Adding value through analysis.
- Making the data easily accessible to others (e.g. downloads, GUIs).

Most databases and their associated tools are developed for research and public health purposes. For these reasons, curators should make downstream analyses derived from the tools and curated databases available. In a sense, this is an additional layer of data that itself requires curation.

Here we describe how we acquire and curate the genes, point mutations, blast rules and HMM models used in NCBI’s AMRFinderPlus [9, 10]. We then describe the steps we take to ensure database quality. Using the Reference Gene Catalog as an example, we detail how the databases, rules and models are made publicly available, as well as how to access the software. We discuss how the computed analysis of those tools can be accessed. Finally, we conclude with NCBI’s plans to make these databases accessible over the long-term and plans for future improvements in our databases.

### AMRFinderPlus and its associated databases as a model

Before we describe how we curate the databases used by AMRFinderPlus, we wish to review several key features of AMRFinderPlus (further details are available in [9, 10]). AMRFinderPlus searches either nucleotide or protein sequence, or both jointly, for acquired stress response, virulence and antimicrobial resistance (AMR) genes, as well as point mutations. Taxon-specific point mutations are identified by blast [11, 12] against a taxon-specific set of reference protein and nucleotide sequences. AMRFinderPlus output provides the position of each element (gene or point mutation), the method used for identification (blast or HMM), and possible phenotypes. Acquired genes are identified either through blast against a reference database, with each gene possessing a manually curated blast cutoff, or, if protein sequence is available, through a combination of blast and hidden Markov models (HMMs), both of which have manually curated cutoffs.

**Fig. 2.**
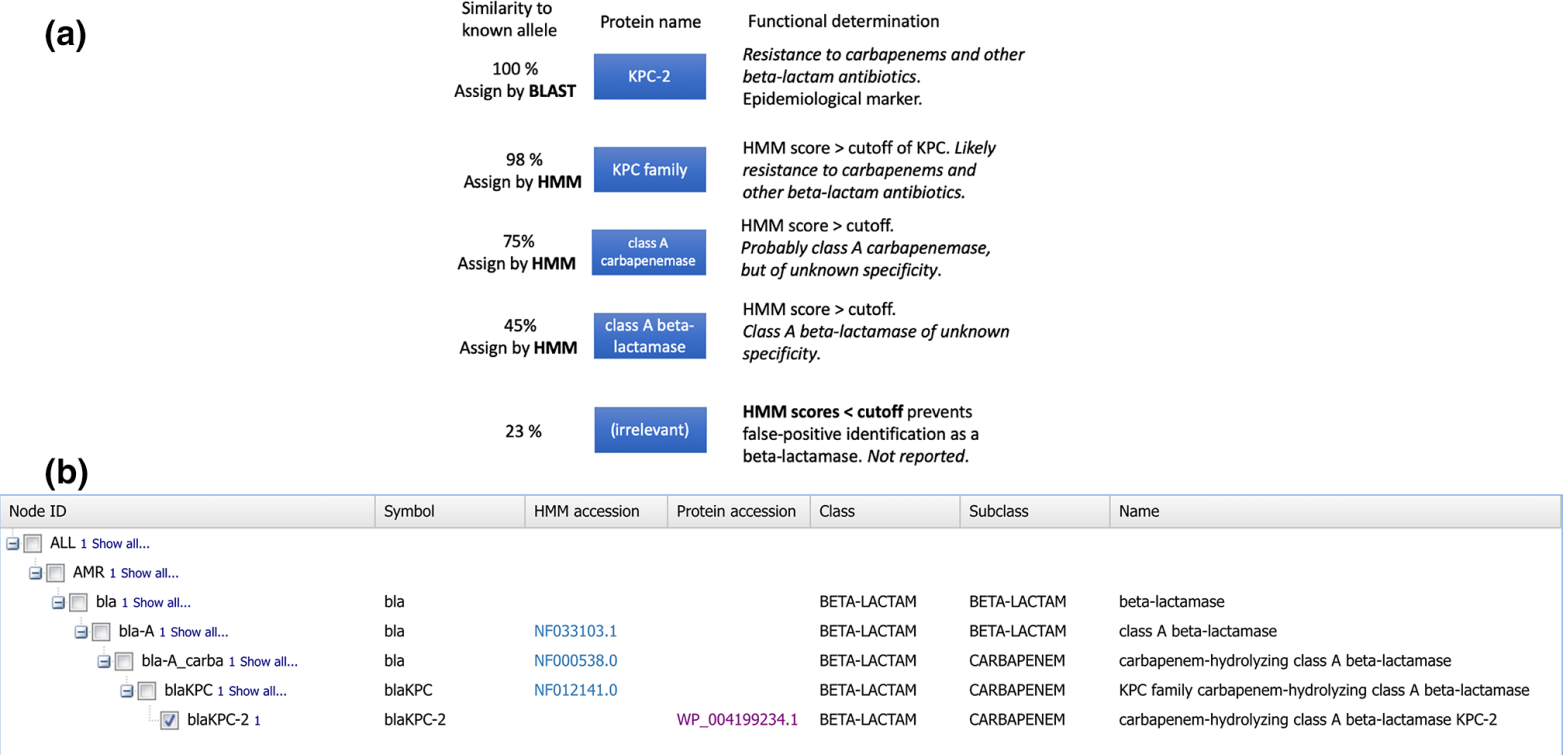
. (a) Example of AMRFinderPlus’ hierarchical structure, starting with *bla*_KPC-2_ at the top and moving to less specific proteins. b) A screenshot showing how *bla*_KPC-2_ is displayed in the Reference Hierarchy Viewers (https://www.ncbi.nlm.nih.gov/pathogens/genehierarchy/#blaKPC-2). Note that the uppermost ‘bla’ row is an organizational node, and lacks a HMM, so it is not represented in (a).

A novel feature of AMRFinderPlus is that genes, which can have one or more proteins, are assigned to a node in a hierarchy (Fig. 2a). For example, conceptually we can view how the classification of a beta-lactamase would work. A protein that is 100 % identical to *bla*_KPC-2_ is clearly *bla*_KPC-2_. A novel, but only slightly divergent protein would be called *bla*_KPC_, and would likely, though not necessarily confer resistance to carbapenems. A somewhat more divergent beta-lactamase would be assigned to a node composed of related class A beta-lactamases (with gene symbol *bla*), while even more divergent proteins could be identified as class A beta-lactamases or even as beta-lactamases of unknown class. This allows AMRFinderPlus to report the most accurate gene name, reflecting possible ambiguity in its functional annotation, as opposed to the name of the nearest gene as defined by sequence identity [13, 14].

Thus, the AMRFinderPlus database has four essential components:

1. An acquired gene database of AMR, stress response and virulence genes. This is a collection of genes (and gene symbols), each of which contains one or more proteins, often with an associated BlastRule (protein identity threshold) or HMM, along with phenotypic data and other descriptive metadata.
2. A collection of point mutations and reference sequences that contains the site(s) of the mutations, the reference sequence and the target organism, along with phenotypic data and other descriptive metadata.
3. A collection of HMMs constructed in HMMER3 [15] with manually curated cutoffs. The raw HMMER3 file is stored, and these HMMs also are integrated into two separate NCBI sources.
4. A gene family hierarchy, which enables the accurate naming and identification of both novel and known protein sequences.

To reflect the constant change in the literature, curators continuously update these databases with new releases made approximately every 2 months.

## Methods

### Curation

Curation starts with the reporting of resistance and virulence mechanisms in the primary scientific literature. As described previously [9, 10], NCBI gathers novel genes and point mutations through a variety of mechanisms, including inter-organizational data exchanges, surveys of the literature, requests from collaborators and domain experts, pre-existing data sources, as well as requests for allele assignment of over 40 families of beta-lactamases, quinolone resistance genes (Qnr), and mobile colistin resistance genes (MCR).

We typically require experimental evidence or an extremely close identity hit to an experimentally verified protein for inclusion of acquired resistance alleles, genes and point mutations that are considered ‘core’ [10]. Curators do not require supporting evidence in the literature for allele assignment as a goal of resistance allele assignment is to encourage characterization and further study of the phenotypes and effects of these individual proteins (alleles are gene symbols that map to only one specific protein sequence) [16]. Here we describe specific features of the curation process for genes, alleles and point mutations.

### Gene curation

For genes, curators will assign product names and a gene symbol (e.g. *bla*_TEM_) and also ensure that the reference sequence is correct based on surveys of the literature and multiple sequence alignments. Then curators will edit the product name of the corresponding RefSeq protein record and identify a suitable RefSeq nucleotide sequence, which is subsequently assigned the curator-determined product name, gene symbol and allele designation. For genes that fall into the plus category – those genes related to biocide and stress resistance, general efflux, virulence, or antigenicity, or AMR genes whose phenotype is uncertain – curators assign product names and gene symbols, but no RefSeq nucleotide records are created.

### Allele curation

When alleles are submitted to NCBI for allele assignment, we work with the submitter to rectify possible sequence problems. Specifically for alleles, curators compare submitted alleles to existing alleles in related families by sequence alignment [11, 12], as well as run AMRFinderPlus on these alleles to determine if the protein is full-length, functional, and if it belongs to an existing family. When a submission meets these requirements, then an automated process confirms that the allele is novel, and sequentially assigns it a new designation, preventing duplicate assignments. On occasion, researchers have assigned themselves alleles, creating conflicts, and NCBI curators attempt to resolve these conflicts.

### Point-mutation curation

For point mutations, curators identify an appropriate reference protein or nucleotide sequence in GenBank, assign a name to be used by AMRFinderPlus, a mutation symbol that contains the location of the point mutation, and a gene symbol for the reference sequence. Point mutations are defined as *individual* differences from the reference. Our inclusion criteria for point mutations requires either experimental validation or strong phenotypic correlations that could not be accounted for by other resistance mechanisms.

### Hierarchy node curation

Following sequence addition to the database, curators will develop either BlastRules or HMMs for a given node in the gene hierarchy. BlastRules are used when a small number of proteins exist for a gene, and novel proteins above a certain manually curated threshold are deemed members of that gene or gene family. If novel BlastRules are created or existing ones are updated due to addition of new sequences, these changes are added to NCBI’s Protein Family Model database [17]. HMMs are built when there are multiple proteins with a gene or gene family, and they provide more evidence and resolution for inclusion within a gene or family. These HMMs are stored in NCBI’s Protein Family Model database for use in PGAP [17]. We have described the details of HMM creation elsewhere [10]. Curators are alerted if there are conflicts between HMM results and the gene hierarchy; for example, curators are notified when a protein is hit by a HMM, but that HMM’s gene family is not considered to be an ancestor of the node to which a protein is assigned.

### Structural annotation

Along with functional annotation to determine if a genetic element should be included, curators assess structural annotation to determine that the sequence of the genetic element is appropriately characterized. For all alleles and genes, NCBI has a series of 54 automated quality-control checks (Table S1, available in the online version of this article) that are run during the release process to ensure the quality of each protein sequence added to the database. These checks serve as an automated backstop for the manual curation process. Some checks examine basic sequence problems, such as partial proteins that are misannotated, proteins with ambiguous bases, stop codons, frame shifts and other errors. Checks may flag proteins for quality reasons, such as partial proteins, determined either by the protein product name containing the word ‘partial’ or by comparison to similar proteins, and proteins with ambiguous residues. These issues often can be resolved by finding alternative sequences or contacting the original sequence submitters. Automatic and manually applied quality-control checks reduce the incidence of these errors. Alleles are automatically checked for possible errors, including ambiguous bases, frameshifts or stop codons.

### Resolving nomenclature conflicts

Nomenclature conflicts and problems in the source literature also can affect gene databases. Multiple authors can use the same gene symbol for different sequences or genes, requiring curators to identify and resolve these conflicts. For example, when mobile colistin resistance genes were first identified, researchers unfortunately submitted different and diverse sequences with colliding gene names. As part of a large consortium, NCBI helped resolve these conflicts [18]. We have multiple checks that can identify such conflicts, summarized here and described in detail in Table S1. In addition, gene symbols are checked to ensure consistency and accuracy, and curators are alerted if gene symbols are non-standard or if they are linked to multiple product names.

### Integration into other NCBI resources

These databases inform and are integrated into NCBI’s annotation tools, such as PGAP [17] and RAPT (https://www.ncbi.nlm.nih.gov/rapt). An automatic system identifies any differences between the implementation of BlastRules and HMMs in AMRFinderPlus and their implementation in NCBI’s Protein Family Model database so curators can harmonize these databases. All of these automated checks ensure that any obvious discrepancies will be reviewed by curators.

### Testing of AMRFinderPlus

**Fig. 3.**
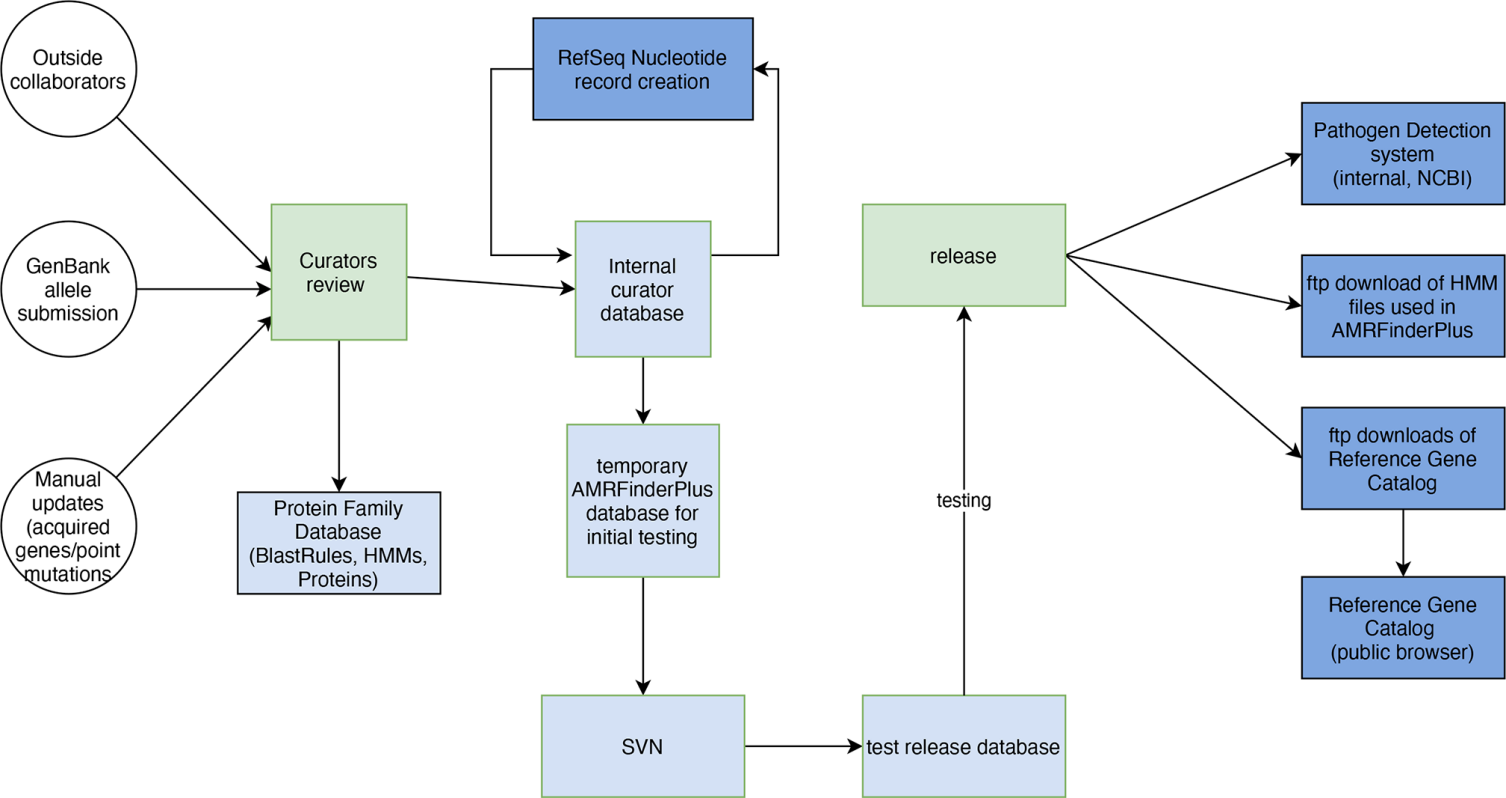
How curators update and release AMR database products. Circles are data sources, squares are processes, and diamonds are tests. Orange is for curator supervised steps, light blue is for internal-only, light green is other NCBI resources and databases, dark blue is for public access to complete data files, and dark green is for public access through web interfaces. ‘SAUTE guided assembler’ refers to a set of non-redundant nucleotide sequences derived from the Refseq nucleotide sequences of acquired AMR genes and some virulence genes deemed of critical importance. These sequences are used by the SAUTE guided assembler in the Pathogen Detection assembly process to ensure assembly of these critical genes.

In addition, before release, developers initiate a series of over 200 tests of AMRFinderPlus using the release candidate database that can detect unwanted errors and highlight potential improvements curators might make (Fig. 3). These tests include processing all sequences from our internal database, all point mutations, a set of real public assemblies, varying software options, and known edge cases. All changes in AMRFinderPlus results from the previous database release are manually reviewed. New release candidates are generated, and all of the above QC tests are performed again until all tests pass and any automated test failures are manually reviewed by curators.

## Results

We have previously validated and published the AMRFinderPlus method, but we summarize briefly here. In one study, we compared the identification of AMR genes and point mutations to known phenotypes of 6242 isolates, and found that 98.4 % of phenotypes were consistent with the genotypic predictions [9]. In a separate study, we assessed the accuracy of the some of the plus genes, specifically metal resistance against a series of mercury-resistant isolates, and found a high correlation between genotypic predictions and observed phenotypes [10].

In addition, database curation is informed by the release of NCBI’s publicly available AMRFinderPlus results. Where possible NCBI participates in nomenclature related collaborations; as described in Methods, NCBI was part of a multi-group effort to standardize mobile colistin resistance gene (MCR) nomenclature [18]. As a result, these updates were incorporated in the database and used in AMRFinderPlus analyses made publicly available through the Isolates Browser and MicroBIGG-E. Another example is a collaboration with the National Antimicrobial Resistance Monitoring System (NARMS) in the U.S. where NCBI Pathogen Detection found that *mcr-9* is the most common MCR variant in the US. NARMS initiated a phenotype study that found *mcr-9* did not confer resistance to colistin in over 100 natural *mcr-9* +isolates [19], despite laboratory evidence that *mcr-9* is capable of conferring resistance to colistin [20, 21]. Based on this finding, curators made the decision to move *mcr-9* to the plus category. This feedback loop between a publicly available database and analysis results and experimental verification is critical for improving our understanding of genotype-phenotype relationships and public health.

The publicly released data also enable analyses to better understand AMR trends and patterns. For example, a recent study used the Isolates Browser, which links AMRFinderPlus output with isolate metadata, to assess the global resistome and evolutionary epidemiology of multiple Enterobacterales species [22].

### Database access and exploration

After new data is incorporated into internal curation tools, the data are released to the public, both as data files and in graphical user interfaces, as well as incorporated into NCBI’s Pathogen Detection system (Fig. 3). AMRFinderPlus is run on over 1 million bacterial isolates and the results of these analyses are released in browsers and other formats. Here, we describe how these databases are made accessible to the public (Fig. 3).

One way for users to access the databases is through data files on our FTP site. These files can be of use for those who want to interrogate the data in detail or who want to use them as part their own data analysis process. From there, users can download the following as tab-delimited text files: the Reference Gene Catalog, the Reference Gene Hierarchy used by AMRFinderPlus, the allele counts of the AMR genes curated by NCBI by year. Users also can download the HMMs used by AMRFinderPlus and release notes describing the most recent curatorial changes. In addition, there are FASTA formatted files containing the protein and nucleotide reference sequences used by AMRFinderPlus.

**Fig. 4.**
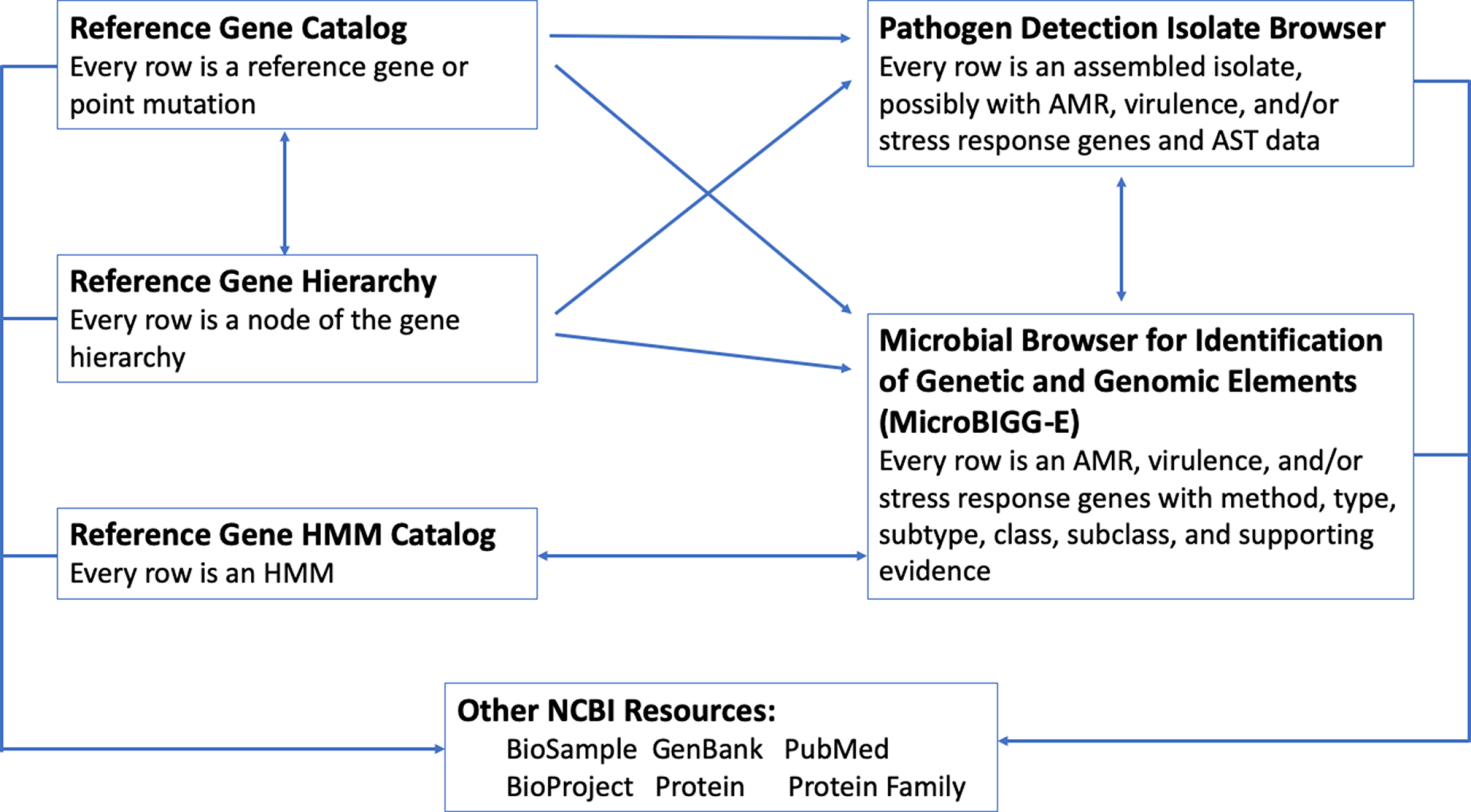
Interactions among database viewers and existing NCBI resources. Arrows represent links resulting from selection of individual field values or cross-browser selection.

NCBI has built multiple browsers to enable viewing and dynamic browsing of these various databases. A key new feature in all of the browsers is the ability to use hyperlinks that let users explore subsets of data identified in one browser in other browsers. Although we have described each of these databases as stand-alone entities, they have been designed to interact with each other, enabling links between the database views and the analytical views (Fig. 4, Table 1). We have described previously how both the Isolate Browser and MicroBIGG-E allow cross-browser selection, whereby sets of isolates or genes selected in one resource or the other allows selections in the other resource, either the set of genes encoded by the isolates, or the set of isolates that encode the genes, respectively [10], and so these will not be discussed here.

**Table 1.** Field-specific links within browsers. ‘Browser’ describes the browser. ‘Field in browser’ describes the specific column with the hyperlink. ‘Function’ describes what is displayed upon selecting the hyperlink

Browser	Field in Browser	Function
**Isolates Browser**	Assembly	Links to Assembly record for that page
	BioSample	Links to BioSample page for that BioSample
	BioProject	Links to BioProject record for that BioProject
**MicroBIGG-E**	Assembly	Links to Assembly record for that assembly
	BioSample	Links to BioSample record for that BioSample
	BioProject	Links to BioProject record for that BioProject
	Closest Reference Accession	Links to Protein record of closest reference protein
	Contig	Links to Nucleotide record of contig containing the element
	HMM Accession	Links to HMM record in Protein Family Model database
	Protein	Links to Protein record
	PubMed ID	Links to related PubMed record(s) for that genetic element
	Start/Stop	Links to Nucleotide record of contig containing the element but displays only the element itself
**Reference Gene Catalog**	Allele	Links to all isolates in the Isolates Browser containing that allele
	Gene Family	Links to all isolates in the Isolates Browser containing that gene family
	GenBank Nucleotide Accession	Links to the GenBank Nucleotide Record for that element
	GenBank Protein Accession	Links to the GenBank Protein Record for that element
	Hierarchy Node ID	Displays that node in the Reference Gene Hierarchy
	PubMed ID	Links to related PubMed record(s) for that genetic element
	RefSeq Nucleotide Accession	Links to the RefSeq Nucleotide Record for that element
	RefSeq Protein Accession	Links to the RefSeq Protein Record for that element
**Reference Gene Hierarchy**	HMM Accession	Links to HMM record in Reference HMM Catalog
	Protein	Links to Protein record
**Reference HMM Catalog**	Accession	Links to HMM record in Protein Family Model database
	MicroBIGG-E	Displays all genetic elements identified by the HMM in MicroBIGG-E

The Pathogen Detection Reference Gene Catalog (https://www.ncbi.nlm.nih.gov/pathogens/refgene/) provides a visualization of acquired genes and point mutations used by AMRFinderPlus. Each row represents an acquired protein sequence or point mutation (see Fig. 1). These fields have hyperlinks that connect to other NCBI databases such as GenBank and PubMed. Users also can use links to display every isolate with that genetic element in the Isolates Browser or to view all instances of that element in MicroBIGG-E (Fig. 3). For example, users in the Reference Gene Catalog can click a hyperlinked gene family symbol (e.g. *aac(3)-I*, and they will be automatically directed to MicroBIGG-E with every instance of *aac(3)-I* displayed. The Reference Gene Catalog also describes additional information about each sequence or point mutations such as the taxa for which detection of that sequence is excluded, information about its phenotype, location on the GenBank contig, a PubMed citation (if available), and other metadata (Fig. 4, Table 1). Users also can download these data in a table format.

The previously undescribed Reference Gene Hierarchy is a web-based view into the hierarchy of genes, families and upstream nodes that NCBI curators use to organize and relate the genes and HMMs in the Reference Gene Catalog and Pathogen Detection Reference HMM Catalog. This hierarchy drives the gene identification and naming algorithm of AMRFinderPlus. The Pathogen Detection Reference Gene Hierarchy provides the link between proteins, HMMs and protein names for those proteins that do not have an exact match in the Reference Gene Catalog. In the Reference Gene Hierarchy Viewer, this can be used (Fig. 2b) to better interpret AMRFinderPlus output and understand the organization of the data. For instance, in the Reference Gene Hierarchy, after searching for ‘*bla*_KPC_’, which returns the entire family of KPC-family carbapenemases (https://www.ncbi.nlm.nih.gov/pathogens/genehierarchy/#blaKPC-2), users can see this family displayed in the Reference Gene Catalog, see every instance of a KPC-family carbapenemase displayed in MicroBIGG-E, or find which isolates in the Isolates Browser have a KPC-family carbapenemase.

The Pathogen Detection Reference HMM Catalog is a new web-based portal to our curated database of reference HMMs used by AMRFinderPlus in concert with gene sequences in the Pathogen Detection Reference Gene Catalog to identify AMR genes as well as some stress resistance and virulence genes. This is a highly curated subset of the HMMs included in NCBI’s Protein Family Models database [17]. Every row in the Pathogen Detection Reference HMM Catalog is an individual HMM. Details including the seed alignment and HMM profile are available by clicking on the HMM accession in the table.

### Computed analyses

In addition to the AMRFinderPlus databases, NCBI also runs AMRFinderPlus on genomes belonging to 47 bacterial taxonomic groups as part of NCBI’s Pathogen Detection project [23]. The Isolates Browser (https://www.ncbi.nlm.nih.gov/pathogens/isolates/) displays a summary of AMR, stress response and virulence genes for each isolate of interest, where each row describes an isolate assembly. When submitted to NCBI antibiotic susceptibility testing data can be linked to each isolate. These data can be downloaded for further analysis. The Microbial Browser for Identification of Genetic and Genomic Elements (MicroBIGG-E; https://www.ncbi.nlm.nih.gov/pathogens/microbigge/), displays AMRFinderPlus results for those isolates that have genomic data deposited in GenBank, displayed in a more comprehensive format resembling the AMRFinderPlus output. In this case each row is a detected gene or point mutation. Information about how it was identified, links to the sequence, phenotype and reference data are included as well as isolate metadata such as BioSample and strain names, and isolate source that are also shown in the Isolates Browser. Sequences and table data can also be downloaded in bulk here. Importantly, both of these views display and store the versions of the software and database that was used to analyse each isolate so users can determine if analyses were run with the database version containing their genes or mutations of interest since new genes and point mutations are added with each release and could affect the computed results. At each release, release notes indicate changes so users can determine if they deem it necessary to rerun AMRFinderPlus themselves with newer database or software version.

These computed analyses have been used successfully by other researchers. For example, one group used the output from MicroBIGG-E to assessing the health risk of antimicrobial resistance genes [24]. These tools also have been used to survey *Acinetobacter* sp. for resistance mechanisms [25]. MicroBIGG-E enables researchers to collect a diverse set of AMR-related sequences, as was done in a study examining the function of erythromycin resistance methyltransferases [26].

## Discussion

Databases constructed for analytical tools are not only part of those tools, but are useful to researchers themselves. Here, we have described four primary components of the AMRFinderPlus database curated by NCBI. Inclusion requires either experimental evidence or strong correlations between genotypes and phenotypes. The database undergoes multiple quality-control checks that ensure data reliability and accuracy. We have also developed multiple online tools that make it easier for researchers to access the databases and examine their structure and data, as well as subdivide the data for further use. These databases are accessible in multiple formats and locations.

One advantage AMRFinderPlus has is that many of its components are integrated into other NCBI-maintained systems. The Protein Family Model database contains both the Blast Rules and HMMs used by AMRFinderPlus, and is the backbone of NCBI’s PGAP tool, which is used for annotation at NCBI [17, 27]. Since all sequences in the Reference Gene Catalog are accessioned in GenBank, these will remain archived. The tight integration of the AMRFinderPlus database components with other NCBI systems along with being an integral component of NCBI’s Pathogen Detection project and a part of the U.S. National Action Plan for Combating Antibiotic-Resistant Bacteria [28] further ensures that the underlying databases and their component data will continue to be available.

While we have an expansive database covering 5940 AMR genes, 914 point mutations, 233 stress response genes and 716 virulence genes, we recognize that there are still significant gaps. One way NCBI has attempted to fill the gaps is through collaborations with outside groups. For example, NCBI worked with multiple U.S. government agencies to add stress response and virulence genes found in food-borne pathogens that are critical for understanding the links among AMR, stress response (e.g. biocides) and virulence [10]. Not only does our database rely on these collaborators as well as the published literature, but we also use publicly available forums such as CARD’s amr_curation discussion group (https://github.com/arpcard/amr_curation) and requests from outside investigators via the Pathogen Detection project (pd-help@ncbi.nlm.nih.gov) to improve and expand the databases. NCBI invites domain experts to contact us if they have requests to support organisms of interest, and we have multiple collaborations to expand and improve databases [18]. We also engage in data harmonization with CARD [29] and other sources.

In collaboration with our interagency partners, we have prioritized food-borne pathogens and high-priority AMR pathogens, so many of the 47 bacterial taxa in the Pathogen Detection system do not have taxon-specific virulence factors. In addition, only 14/47 taxa currently have point mutations, and AMRFinderPlus currently does not cover *Mycobacterium tuberculosis* point mutations at all. These are all directions for future work to improve the AMRFinderPlus database. Given the importance of bioinformatic approaches to combatting the problem of AMR, we hope that these databases will assist other researchers in their own efforts to combat this problem.

## Supplementary Data

Supplementary material 1
